# Association Between Maternal Characteristics and the Risk of Isolated Maternal Hypothyroxinemia

**DOI:** 10.3389/fendo.2022.843324

**Published:** 2022-04-12

**Authors:** Yang Liu, Guohua Li, Nafei Guo, Xiaosong Liu, Shijia Huang, Qiaoling Du

**Affiliations:** ^1^Department of Obstetrics, Shanghai Key Laboratory of Maternal Fetal Medicine, Shanghai First Maternity and Infant Hospital, School of Medicine, Tongji University, Shanghai, China; ^2^Department of Reproductive Immunology, Shanghai Key Laboratory of Maternal Fetal Medicine, Shanghai First Maternity and Infant Hospital, School of Medicine, Tongji University, Shanghai, China; ^3^Department of Nursing, Shanghai Key Laboratory of Maternal Fetal Medicine, Shanghai First Maternity and Infant Hospital, School of Medicine, Tongji University, Shanghai, China

**Keywords:** isolated maternal hypothyroxinemia, maternal characteristics, risk factor, fetal neurodevelopment, iron deficiency

## Abstract

**Objective:**

We aimed to determine the association between maternal characteristics and isolated maternal hypothyroxinemia (IMH).

**Methods:**

Pregnancies registered at Shanghai First Maternity and Infant Hospital between January 2014 and September 2020 were included in this cross-sectional study. IMH was defined as free thyroxine (FT4) levels below the 10th percentile with TSH within the normal reference range. Multivariate logistic regression models were used to identify potential risk factors for IMH, including demographic information, anthropometric measurements and nutritional status.

**Results:**

A total of 54586 singleton pregnancies were included, involving 6084 women with IMH and 48502 euthyroid women. Multivariate logistic regression analyses showed that the variables for women with ages ≥35 (adjusted OR = 1.30, 95% CI:1.20–1.40), non-local residence (adjusted OR = 1.16, 95% CI:1.09–1.23), multiparas (adjusted OR = 1.11, 95% CI:1.03–1.21), pre-pregnancy overweight (adjusted OR = 1.37, 95% CI:1.27–1.49) or obesity (adjusted OR = 1.35, 95% CI:1.18–1.54), and iron deficiency (adjusted OR = 1.27, 95% CI:1.20–1.35) were independent risk factors for IMH in the overall study population, which were identical to those in the first trimester subgroup.

**Conclusions:**

Maternal characteristics were associated with the onset of IMH. Maternal age, residence of origin, parity, pre-pregnancy body mass index (BMI) and iron status should be comprehensively considered to evaluate the risk of IMH, according to which obstetricians could determine an optimal assessment time for thyroid function.

## Introduction

Thyroid hormone (TH) is crucial for normal fetal development and increases by approximately 50% during pregnancy (1), guaranteeing the proliferation and migration, as well as other developmental processes of fetal brain cells (2). It has been proven in animal studies that the lack of TH at an early stage of pregnancy leads to severe brain defects (3, 4). Isolated maternal hypothyroxinemia (IMH) is a kind of mild thyroid insufficiency characterized by low free thyroxine (FT4) but normal thyroid-stimulating hormone (TSH) levels in pregnant women. Some epidemiological studies have shown that IMH diagnosed in the first trimester is not only associated with impaired mental and motor neurodevelopment, such as lower IQ levels (5) and slower response speeds (6), but also higher risks for psychiatric disorders of the offspring (7–9). In addition to the effect on fetal neurodevelopment, IMH may also increase the risk of obstetric complications, such as preterm delivery, hypertension disorders, and gestational diabetes (10).

The etiology of IMH is not fully understood. Iodine deficiency is theoretically recognized as a major factor because the insufficient supply of iodine would probably lead to a prior production of T3 to T4 (11). However, some studies from iodine-deficient areas have shown conflicting results that there was no difference in urinary iodine concentration (UIC) between women with IMH and euthyroid pregnant women (12, 13). Furthermore, iron deficiency (ID) has recently been raised as another potential risk factor for IMH because women with ID had a significantly higher prevalence of hypothyroxinemia than those without in two independent cohorts (14, 15). Other factors, including obesity, environmental pollution, and angiogenic factors, were also found to be associated with lower FT4 levels, but their role in IMH remains uncertain (16–18).

It is believed that the detrimental effect of IMH on fetal neurodevelopment can be prevented or rescued by timely levothyroxine administration (3), and the ideal treatment should be ahead of the sixth week of gestation, at which time early neurogenesis starts. Currently, the first obstetric visit in China is commonly set at the late stage of the first trimester, which is too late for optimal treatment to ensure normal fetal brain development. Therefore, an early identification of IMH risk factors based on maternal characteristics seems to be preferable and reliable. However, there were limited studies comprehensively estimated the potential risks from this perspective.

The main aim of this study was to investigate the association between maternal characteristics and the risk of isolated hypothyroxinemia in a large obstetric care center in Shanghai, where iodine status was defined as adequate (19, 20).

## Material and Methods

### Study Population

**Figure 1:**
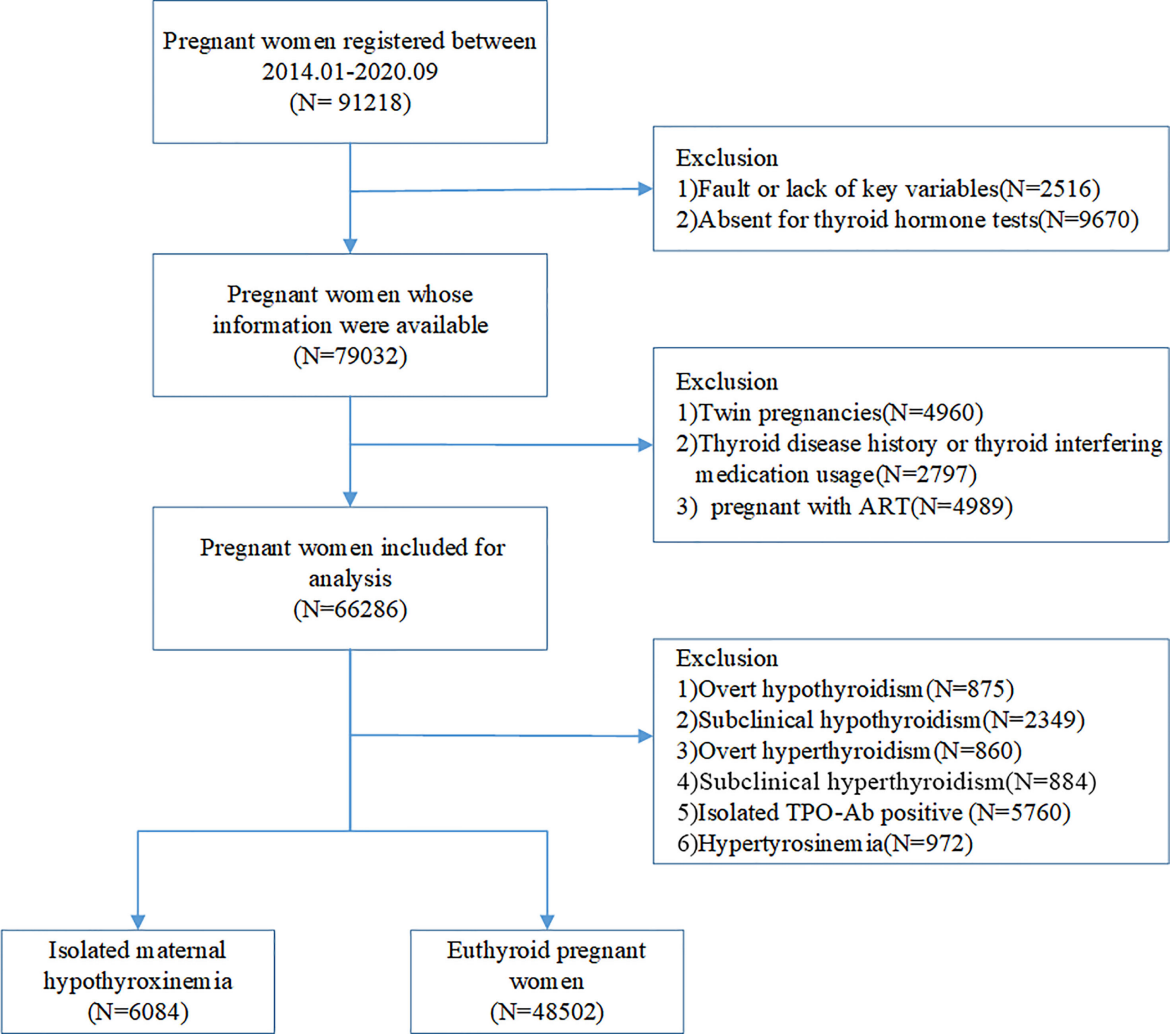
Flow chart for study population.

This is a cross-sectional study based on electronic medical registers. A total of 91812 pregnant women who underwent antenatal health care and planned to deliver at Shanghai First Maternity and Infant Hospital between January 2014 and September 2020 were included, from which 2516 were excluded due to a fault or lack of key variables, and 9670 for not receiving thyroid hormone tests. The exclusion criteria for this study were as follows: 1) women with a twin pregnancy; 2) women with a history of thyroid disease, including overt or sub-clinical hyperthyroidism, hypothyroidism, and thyroid cancer; 3) women who used thyroid interfering medication, including levothyroxine, glucocorticoids, and rifampicin; 4) women who got pregnant using assisted reproductive technology (ART). After the exclusion of women diagnosed with overt hypothyroidism (n=875), subclinical hypothyroidism (n=2349), overt hyperthyroidism (n=860), subclinical hyperthyroidism (n=884), hyperthyroxinemia (n=972) and isolated TPOAb positivity (n=5760) during pregnancy, we chose women with isolated maternal hypothyroxinemia (n=6084) and unaffected pregnancies (48502) as the final study population (**Figure 1**). The study was approved by the Ethics Committee of the Shanghai First Maternity and Infant Hospital.

### Maternal Characteristics and Medical History

Demographic information, including ethnicity, age, parity, BMI before pregnancy, medical history, and medication usage, was obtained through questionnaires. Information on obstetric outcomes, date of birth, and fetal sex was obtained from discharge records. Anthropometric measurements, including height and weight, were performed when participants were recruited and were used to calculate the pre-pregnancy BMI.

### Laboratory Measurements

Maternal venous serum samples were obtained for the measurements of FT4, TSH, TPOAb, serum ferritin (SF), and 25-hydroxyvitamin D. FT4, TSH and TPOAb were measured using a chemiluminescence immunoassay (ADVIA Centaur, Siemens Healthcare Diagnostics). The minimum detectable concentrations of FT4, TSH and TPOAb were 1.3 pmol/L, 0.005 mIU/L, and 28 U/mL, respectively. The intra-assay coefficients of variation were 3.3%, 2.2%, and 2.5% at FT4 concentrations of 9.3, 19.0, and 38.8 pmol/L, respectively; 2.3%, 2.9%, and 4.5% at TSH concentrations of 0.60, 30.3, and 109 mIU/L, respectively; and 6.8% and 1.3% at TPOAb concentrations of 70.8 and 458.5 U/mL, respectively. SF measurements were carried out using a UniCel DxI 800 immunology analyzer and kits (Beckman Coulter, California, America). The intra-assay coefficients of variation were 2.6%, 3.6%, and 3.9% at ferritin concentrations of 37.2, 118.9, and 311.8 ng/mL, respectively. 25-hydroxyvitamin D levels were analyzed using a chemiluminescence particle immunoassay (Architect Alinity, Longford, Ireland). The intra-assay coefficients of variation were 2.2%, 2.1%, and 1.9% at 25-hydroxyvitamin D concentrations of 20, 39.7, and 75.6 ng/mL respectively.

### Definition

We built a population-based, trimester-specific reference range for FT4 and TSH levels according to the guideline (21) (**Table S1**). Isolated maternal hypothyroxinemia was defined as FT4 levels below the 10th, the 5th and the 2.5th percentile respectively based on previous studies and the ATA guideline (22), with TSH levels within the reference range in the present study. Based on the manufacturer-defined cutoff, iron deficiency was defined as serum ferritin lower than 12 µg/L, and TPOAb > 60 IU/ml was considered positive. Women over 35 years old was defined as advanced age. The non-local residence was defined as participants who were not born or settled permanently in Shanghai. Women who did not belong to the Han population were defined as ethnic minorities. BMI was classified into four groups according to the reference range for Asian populations: <18.5 kg/m^2^ (underweight), 18.5 to <23 kg/m^2^ (normal), 23 to <27.5 kg/m^2^ (overweight), and ≥ 27.5 kg/m^2^ (23). All the covariates were further described in **Table S2**.

### Statistical Analyses

Histogram plotting was used to evaluate the data distribution. Continuous variables with a normal distribution are presented as the mean ± SD (Standard Deviation) and were compared between two groups using Student t-test. Skewed distributed variables are presented as medians (interquartile ranges) and were analyzed with the Wilcoxon test. Categorical variables are presented as cases (percentages), and the chi-square test was used for comparisons between two groups. The primary analysis was performed using the 10th percentile definition to include more subjects and find their similar characteristics. Multivariate logistic regression models were used to identify potential risk factors for IMH, and Ln-transformed TSH and average 25-OH-D concentration were included as continuous variables in the model. The 2.5th and 5th percentile cut-offs were used respectively in this analysis since these cut-offs were also widely used. All statistical analyses were conducted in SAS 9.4 (SAS Institute Inc., Cary, NC, USA). A *P value* < 0.05 was considered statistically significant.

## Results

The study population had a mean (SD) age of 30.21 (3.75) years and BMI of 21.08 (2.74) kg/m2. The prevalence of IMH was 10.21%, 10.58%, and 9.9% for the first, second and third trimesters, respectively.

Compared with euthyroid women, women with IMH were more likely to have advanced age (17.49% vs. 12.61%, *P* < 0.001), prepregnant overweight or obesity (18.56% vs. 14.20%, *P* < 0.001), be from a non-local population (72.47 vs. 68.39%, *P* < 0.001), be multiparas (31.10 vs. 24.79%, *P <*0.001), have higher TSH levels (1.50 vs. 1.27, *P*<0.001), and have decreased serum ferritin levels (23.25 vs. 26.08, *P*<0.001) (**Table 2**). There was no difference in ethnicity, fetal sex or 25-hydroxyvitamin D levels between the two groups (**Table 1**).

**Table 1:** Univariate analysis of maternal characteristics and metabolic parameters between euthyroid women and women with isolated hypothyroxinemia.

Characteristics	Euthyroid	IMH	*P*
**Age (mean ± SD)**	**30.126 ± 3.724**	**30.838 ± 3.904**	**< 0.001**
<35 yrs (N,%)	42384(87.39)	5020(82.51)	
≥35 yrs	6118(12.61)	1064(17.49)	
**BMI (mean ± SD)**	**21.018 ± 2.717**	**21.583 ± 2.851**	**< 0.001**
Optimal (N,%)	34606(71.35)	4312(70.87)	
Underweight	7009(14.45)	643(10.57)	
Overweight	4985(10.28)	850(13.97)	
Obesity	1902(3.92)	279(4.59)	
**Nonlocal (N,%)**	**33170 (68.39)**	**4409 (72.47)**	**< 0.001**
Ethnic Minority (N,%)	1303 (2.69)	192 (3.16)	0.035
**Multipara (N,%)**	**12022 (24.79)**	**1892 (31.10)**	**< 0.001**
**Gravidity >1 (N,%)**	**21789 (44.92)**	**3129 (51.43)**	**< 0.001**
Male fetus (N,%)	25693 (52.97)	3256 (53.52)	0.423
**TSH**	**1.27(0.78–1.85)**	**1.50(0.99–2.10)**	**<0.001**
25-Hydroxyvitamin D	45.65(34.80–58.30)	45.54(35.30)	0.689
**Serum Ferritin**	**26.05(16.70–39.60)**	**23.25(14.70–35.50)**	**<0.001**

IMH, isolated maternal hypothyroxinemia; BMI, Body Mass Index; TSH, thyroid-stimulating hormone.

Bold means these values were statistically significant.

Multivariate logistic regression analysis showed that variables for advanced age (OR = 1.30, 95% CI:1.20–1.40), being a non-local resident (OR = 1.16, 95% CI:1.09–1.23), being multiparas (OR = 1.11, 95% CI:1.03–1.21), having prepregnant overweight (OR = 1.37, 95% CI:1.27–1.49) or obesity (OR = 1.35, 95% CI:1.18–1.54), and having iron deficiency (OR = 1.27, 95% CI:1.20–1.35) were independent risk factors for IMH when the cut-off was defined as 10th percentile. Meanwhile advanced age (OR = 1.34, 95% CI:1.16–1.44), being a non-local resident (OR = 1.22, 95% CI:1.12–1.33), being multiparas (OR = 1.15, 95% CI:1.03–1.29), having prepregnant overweight (OR = 1.47, 95% CI:1.32–1.63), and having iron deficiency (OR = 1.29, 95% CI:1.20–1.40) were independent risk factors for IMH when defined as FT4 lower than 10th percentile. And for IMH set as the lowest 2.5th percentile FT4 levels, advanced age (OR = 1.35, 95% CI:1.21–1.50), being ethnic minority(OR = 1.43 95% CI:1.09–1.87), being a non-local resident (OR = 1.21, 95% CI:1.07–1.36), being multiparas (OR = 1.26, 95% CI:1.08–1.48), having prepregnant overweight (OR = 1.55, 95% CI:1.34–1.79), and having iron deficiency (OR = 1.29, 95% CI:1.16–1.44) were independent risk factors. Ethnicity, fetal sex, and the level of 25-Hydroxyvitamin D were not associated with hypothyroxinemia (**Table 2**).

**Table 2:** Multivariable logistic regression analysis to demonstrate the association between maternal characteristics and hypothyroxinemia.

	IMH(<10%)		IMH(<5%)		IMH(<2.5%)	
	aOR*	*P*	aOR*	*P*	aOR*	*P*
**Advanced age (≥35)**	**1.30(1.20–1.40)**	**<0.001**	**1.35(1.21-1.50)**	**<0.001**	**1.34(1.16-1.44)**	**<0.001**
Ethnic minority	1.12(0.96–1.32)	0.143	1.18(0.96-1.46)	0.117	**1.43(1.09-1.87)**	**<0.001**
**Non-local resident**	**1.16(1.09–1.23)**	**<0.001**	**1.22(1.12-1.33)**	**<0.001**	**1.21(1.07-1.36)**	**<0.001**
**Multiparas**	**1.11(1.03–1.21)**	**0.009**	**1.15(1.03-1.29)**	**0.013**	**1.26(1.08-1.48)**	**<0.001**
**Prepregnant underweight**	**0.76(0.69–0.83)**	**<0.001**	**0.77(0.68-0.87)**	**<0.001**	**0.73(0.61-0.87)**	**<0.001**
**Prepregnant overweight**	**1.34(1.24–1.46)**	**<0.001**	**1.47(1.32-1.63)**	**<0.001**	**1.55(1.34-1.79)**	**<0.001**
**Prepregnant obesity**	**1.35(1.18–1.54)**	**<0.001**	1.18(0.98-1.41)	0.197	1.09(0.85-1.42)	0.701
Male fetus	1.01(0.96–1.07)	0.637	1.06(0.97-1.15)	0.111	1.11(0.99-1.23)	0.054
**Iron deficiency**	**1.27(1.20–1.35)**	**<0.001**	**1.29(1.20-1.40)**	**<0.001**	**1.29(1.16-1.44)**	**<0.001**
Vitamin D level^&^	1.00(1.00–1.00)	0.402	1.00(0.99-1.00)	0.326	1.00(0.99-1.00)	0.990

*All variables in the table were included in the regression model; ^&^average concentrations were used for women with more than one test; OR, odds ratio; SE, standard error.

Bold means these values were statistically significant.

We performed stratified analysis by the assessment time of thyroid function to further determine risk factors for IMH in different trimesters (**Table 3**). For the first trimester, the variables for increased age (adjusted OR = 1.15, 95% CI:1.04–1.27), nonlocal residence (adjusted OR = 1.14, 95% CI:1.06–1.24), multiparas (adjusted OR = 1.26, 95% CI:1.13–1.40), prepregnant overweight (adjusted OR = 1.28, 95% CI:1.15–1.42), prepregnant obesity (adjusted OR = 1.21, 95% CI:1.02–1.45), and iron deficiency (adjusted OR = 1.30, 95% CI:1.21–1.40) were associated with a higher risk for IMH, which was similar to the overall analysis. In the second trimester, except for minority participants (adjusted OR= 1.26, 95% CI: 1.01–1.57), the estimates were in line with those in the first trimester; however, most of the estimates were insignificant in the third trimester.

**Table 3:** Association between maternal risk factors and IMH in different trimesters using multivariable logistic regression analysis.

	First Trimester		Second Trimester		Third Trimester	
	aOR	*P* value	aOR	*P* value	Adjust OR	*P* value
Advanced age	**1.15(1.04–1.27)**	**0.008**	**1.50(1.32–1.70)**	**<0.001**	**1.71(1.16–2.53)**	**0.007**
Ethnic minority	1.06(0.85–1.32)	0.617	**1.26(1.01–1.57)**	**0.043**	0.97(0.43–2.15)	0.934
Nonlocal residence	**1.14(1.06–1.24)**	**0.001**	**1.17(1.06–1.30)**	**0.002**	1.13(0.81–1.57)	0.478
Multipara	**1.26(1.13–1.40)**	**<0.001**	1.09(0.96–1.24)	0.170	0.63(0.41–0.96)	0.031
Prepregnancy underweight	**0.74(0.66–0.83)**	**<0.001**	**0.82(0.71–0.95)**	**0.007**	**0.46(0.24–0.90)**	**0.023**
Prepregnancy overweight	**1.28(1.15–1.42)**	**<0.001**	**1.40(1.21–1.61)**	**<0.001**	1.72(0.85–3.49)	0.541
Prepregnancy obesity	**1.21(1.02–1.45)**	**0.032**	**1.51(1.22–1.86)**	**<0.001**	**0.87(0.56–1.36)**	**0.027**
Male fetus	1.04(0.97–1.12)	0.232	0.99(0.90–1.08)	0.740	1.00(0.75–1.32)	0.983
Iron deficiency	**1.30(1.21–1.40)**	**<0.001**	**1.29(1.18–1.41)**	**<0.001**	1.02(0.76–1.37)	0.899
Vitamin D level	1.00(1.00–1.00)	0.675	1.00(1.00–1.00)	0.446	1.00(1.00–1.00)	0.172

aOR, adjusted odds ratio; SE, standard error.

Bold means these values were statistically significant.

## Discussion

In the present study, we used the information on maternal characteristics, which were not restricted to the course of pregnancy, to explore the risk factors for IMH. We found that the variables for advanced maternal age, non-local residence, multiparas, pre-pregnancy overweight or obesity, and ID were independent risk factors for IMH.

Fetal thyroid does not start to exert secretion function until 16–20 weeks of gestation, before which time all the hormones needed rely on maternal transmission. The insufficient of thyroid hormone during this crucial period may lead to long-lasting and irreversible damage to fetal brain morphology and neurocognitive development in offspring (5–10). Prompt treatment with thyroxine has been proven effective to rescue the damage from maternal hypothyroxinemia in the early days of pregnancy in animal studies (3), but levothyroxine treatment initiated at 8-20 weeks of gestation did not reduce the intellectual disability in offspring of mild maternal TH insufficiency in clinical studies (24), which means the identification of IMH as early as possible is necessary and urgent.

In previous studies, Etemadi et al. (12) observed a 1.6-fold increased risk of isolated hypothyroxinemia in pregnant women older than 30 years, 1.72-fold increased risk for multiple prior pregnancies, 1.57-fold increased risk in women in rural areas, and 3.34-fold increased risk in capital provinces in Iran. Yu and colleagues found that increased BMI was associated with a higher prevalence of mild and severe hypothyroxinemia in pregnant women, and advanced age was associated with a greater risk of mild but not severe hypothyroxinemia in nonpregnant women of reproductive age (25). In Tengs’ study (15), maternal age and increased BMI were risk factors for hypothyroxinemia in the first and second trimesters, but the inclusive predictors were no longer significant in the third trimester. However, maternal factors included in these studies were not comprehensive mainly because the screening value for IMH was not their core research aim.

Increased age and parity were significantly related to lower thyroxine levels in our study, which was consistent with Etemadis’ findings (12). The underlying mechanism has not yet been completely elucidated, but an age-dependent decrease in serum T4 concentration has been reported in a large-population study (26). This decrease may result from changes in thyroid gland function or peripheral TH metabolism (27), but there are also conjectures that decreased iodine storage is the major cause of hypothyroxinemia in older women and multipara women (12, 28). BMI has been found to be inversely related to FT4 levels in pregnant women in previous studies (29, 30), and an obvious increase in IMH prevalence was observed when the BMI crossed 24 kg/m^2^ during early pregnancy in Hans’ research (16). As an increase in T3 could be seen in association with low T4 levels, some researchers speculated that more T4 was stimulated to convert to T3 by fat-derived leptin (31). It is worth noting that the effect of overweight and obese was also observed in non-pregnant groups, including males and females. Pregnant women from other regions of China had a slightly higher chance of being diagnosed with IMH in our research, which may be a result of long-term iodized salt promotion because Shanghai was classified as an iodine-sufficient/excessive region in the latest two national surveys (19, 20).

We also determined the association between nutritional status, including serum ferritin levels and vitamin D levels, and the risk of IMH, finding that ID was 1.27 times more likely to be diagnosed as IMH in our study. ID has been reported to be associated with lower T4 levels or a higher prevalence of IMH in pregnant women in other studies (14, 15, 32). Iron deficiency is known to have multiple effects on the thyroid axis and importantly affects thyroid hormone synthesis by reducing the activity of heme-dependent thyroid peroxidase (33). There was no difference in serum vitamin D levels between the IMH and euthyroid groups in our study. It has been reported that vitamin D levels have inverse relationships with thyroglobulin antibodies, and therefore may be an indicator of Hashimoto’s thyroiditis. Vitamin D is thought to affect thyroid function via the immune system and its role in the infection process, and there is still a lack of evidence supporting that vitamin D has a direct effect on thyroid hormone metabolism (34).

There are several limitations in our study. First, we did not elucidate the potential impact of iodine deficiency on our results due to a lack of urinary iodine concentration data. However, as previous studies showed, the causal effects of UIC on isolated hypothyroxinemia are still controversial, amid which several studies observed no direct association between UIC levels and the prevalence of IMH, neither other researcher found alterations in maternal thyroxine levels after iodine supplementation in areas with mild to moderate iodine deficiency. Furthermore, given that iodized salt use has been mandatory in China for decades, other potent forms of iodine supplementation would be hard to implement even though a slight influence was found. Second, we measured FT4, rather the FT4 index, to assess maternal T4 when defining IMH, which might lead to marginal biases in reflecting the real prevalence of IMH in the population. However, we used method-specific and trimester-specific reference ranges, as recommended by the American Thyroid Association (21), to control the biases as much as possible. Third, we didn’t describe the relation between the maternal characteristics and pregnancy outcomes via IMH, which would be more convictive for their screening potentials, but limited to the nature of our research method it was hard to determine the causality among the three variables.

## Conclusion

Our study suggested that maternal characteristics were associated with the onset of IMH, including age, area of residence, parity, pre-pregnancy BMI and iron status. Obstetricians should comprehensively evaluate the risk of IMH and then determine an optimal assessment time of thyroid function.

## Data Availability Statement

The original contributions presented in the study are included in the article/**Supplementary Material**. Further inquiries can be directed to the corresponding author.

## Author Contributions

YL researched data and wrote the manuscript. SH, XL, NG, and GL researched data. QD oversaw data collection and analysis, contributed to writing of the manuscript, and reviewed and edited the manuscript. All authors were involved in writing of the paper and had final approval of the submitted and published versions.

## Funding

This work was supported by the research grants from the Health and Family Planning Committee of Pudong New Area (approval number: PW2019D-9) and the Shanghai Science and Technology Commission (approval number: 20Y11907900).

## Conflict of Interest

The authors declare that the research was conducted in the absence of any commercial or financial relationships that could be construed as a potential conflict of interest.

## Publisher’s Note

All claims expressed in this article are solely those of the authors and do not necessarily represent those of their affiliated organizations, or those of the publisher, the editors and the reviewers. Any product that may be evaluated in this article, or claim that may be made by its manufacturer, is not guaranteed or endorsed by the publisher.
