# Supplementary material for: Association Between Maternal Characteristics and the Risk of Isolated Maternal Hypothyroxinemia
Source: Front Endocrinol (Lausanne). 2022 Apr 12;13:843324. doi: 10.3389/fendo.2022.843324 (PMC9039333; doi:10.3389/fendo.2022.843324)
Supplement: Supplementary file 1 [file DataSheet_1.docx]

Table S1. Trimester-specific reference range for TSH and FT4 levels

|  | First trimester | Second trimester | Third trimester |
| --- | --- | --- | --- |
| FT4 (pmol/L) |  |  |  |
| P2.5 | 13.12 | 11.98 | 10.53 |
| P5 | 13.61 | 12.45 | 11.05 |
| P10 | 14.25 | 13.03 | 11.64 |
| P97.5 | 22.53 | 20.7 | 18.58 |
| TSH (mIU/L) |  |  |  |
| P2.5 | 0.05 | 0.02 | 0.16 |
| P97.5 | 3.5 | 3.73 | 4.42 |

Table. S2 Covariate (risk factor) value description

| Covariate | Value |
| --- | --- |
| Age | Continuous variable/Whether the pregnant woman is of advanced age (≥35 years old) |
| Residence | Shanghai/Other regions |
| Ethnic group | ethnic minorities/Han population |
| Prepregnant BMI | Underweight/regular/overweight/obese |
| Parity | Primipara/multipara |
| Biochemical index detection gestational week | First trimester/second trimester/third trimester |
| Vitamin D（VitD） | Continuous variables/whether sufficient |
| Serum ferritin（SF） | Continuous variables/whether sufficient |
| Thyroid Stimulating Hormone（TSH） | Continuity variable (Ln conversion) |
| Fetal sex | Male/female |
